# Looking Toward the Future of Integrated Care: History, Developments, and Opportunities

**DOI:** 10.1007/s11414-024-09894-3

**Published:** 2024-07-10

**Authors:** Ronald W. Manderscheid, Amy Ward

**Affiliations:** 1 Bloomberg School of Public Health, Johns Hopkins University, 10837 Admirals Way, Potomac, MD 20854-1232 USA; 2 Suzanne Dworak Peck School of Social Work, University of Southern California, Los Angeles, USA; 3 East Central University, Ada, OK USA

**Keywords:** Integrated care, Early developments in integrated care, History of integrated care, Future of integrated care, Primary care, Social care

## Abstract

For almost five decades, the development and implementation of integrated care—the simultaneous combination of primary care with mental health and substance use care—has been a major challenge for the behavioral health care field. Integrated care is exceptionally important because many people with behavioral health conditions also have chronic physical health conditions. Early research findings in the mid-1980s showed that persons with mental illness are likely to develop chronic physical conditions earlier and more severely than other people. These findings precipitated efforts to understand this problem and to develop further appropriate integrated care solutions. Subsequently, the US Surgeon General made care integration a major focus of his landmark 1999 *Report on Mental Health*, as did the 2008 Mental Health Parity and Addiction Equity Act and the 2010 Patient Protection and Affordable Care Act. However, it was not until 2014, and later, that integrated care actually began to be implemented more broadly. This article reviews these major developmental milestones, examines current activities, and explores likely developments over the next several years. Major current issues include the response to the COVID-19 pandemic, adjusting to its effects on the behavioral health care workforce, and the growing realization that behavioral health care must address the social determinants of life. Likely developments over the next several years will include devising ways to address our workforce crisis, developing effective community interventions, and implementing population health management strategies; implementing the CMS Innovation in Behavioral Health Model; improving reimbursement practices; and exploring the potential of AI for integrated care. Implications for future service organization and training of behavioral health care providers also are discussed. Granted the severity of the current workforce crisis in behavioral health care, urgent efforts are needed to advance the deployment of integrated care in the short-term future.

## Introduction

Because it relates so closely to efforts to address chronic disease and premature mortality, integrated care—the simultaneous combination of primary care with mental health and substance use care by specialists in each of these fields—has assumed exceptional importance in the behavioral health care field during the past 50 years. This article recounts this history, current implementation steps, and likely future developments.

## Historical View

For more than a century, research has shown that people with mental health or substance use conditions are also likely to suffer from chronic physical problems.^1^ However, in the early decades of the twentieth century, neither general practitioners nor psychiatrists had appropriate training to deal with such comorbidities. Therefore, it was not until the latter half of the twentieth century that significant work actually was undertaken on this issue.

Integrated behavioral health care was originally introduced in the United States in the 1970s. Two very early examples of this introduction include the New York Gouverneur Health Services Program^2^ and the Community Health Center Program of Greater New Haven^3^. Both the Governor and Community health centers were early examples of federally qualified health centers that extended their services to include behavioral health care.^2,3^ To this day, both of these programs continue this important mission.

Research conducted at the National Institute of Mental Health (NIMH) in 1986^4^ documented very clearly that persons with mental health conditions are not only more likely to have chronic physical conditions, but that these conditions occur earlier and with greater severity. This set of findings supported modern work on addressing such comorbidities simultaneously. At almost the same time, another NIMH paper^5^ sought to provide a definition and framework for the role of a primary care physician as a care coordinator for people with serious mental illness. Thus, already in the mid 1980s, the concept of integrated care meant the effort to offer mental health and primary care services simultaneously when warranted.^6^

The next major advance in this work was the production of a Surgeon General’s *Report on Mental Health* in 1999.^7^ In this report, Surgeon General Dr. David Satcher defended the science and practice base of mental health services and also proposed that the next decade of work in the mental health services field would be the implementation of integrated mental health and primary care. Shortly thereafter, in 2000, he held a major conference at the Carter Center in Atlanta, Georgia, to define the next steps that the federal government and private sector should take to implement integrated care.^8^ Conference participants were enthusiastic about this work and looked forward to these developments. Because of these landmark efforts by Surgeon General Satcher, he became a national champion for integrated care. His efforts during the first decade of the twenty-first century and beyond have played a key role in subsequent developments.

Shortly after the Carter Center Conference, the Deputy Surgeon General, Dr. Kenneth Moritsugu, was assigned to identify implementation steps that could be taken collaboratively by the Health Resources and Services Administration (HRSA), the Substance Abuse and Mental Health Services Administration (SAMSHA), the Agency for Health Care Research and Quality (AHRQ), and the Centers for Medicare and Medicaid Services (CMS). Personal recollections from the senior author indicate that the major outcome of the Deputy Surgeon General’s effort was his withdrawal from this effort about two years later because he felt no progress could be made in implementing integrated care through interagency collaboration. Involved agencies feared that their own programs would disappear if they collaborated on integrated care.

During this period, the senior author participated in the activities of the Surgeon General’s Office; he not only helped to organize the conference at the Carter Center, but also collaborated with the Deputy Surgeon General in seeking to promote interagency collaboration. Many different people and organizations were involved in these activities between 2000 and 2005; however, broad-based implementation of integrated care did not begin until more than a decade later.

Throughout this preliminary period, an important practical factor impeded the implementation of integrated care. Frequently, integrated care would be disallowed by both public and private payers since they asserted that a person could only receive a single visit per day. Integrated care represented at least two visits in 1 day.^9^ Even though major advocacy efforts were undertaken with CMS to change this limitation, including by Former Representative Patrick Kennedy, many public and private accounting systems persisted in disallowing integrated care because it represented two visits. It is only very recently, especially in the COVID-19 period, that this practice has begun to change on a large scale.^10^

During this same period, in 2001 and 2002, the concept of integrated care was broadened to include not only mental health and primary care but also substance use care. This resulted in SAMSHA undertaking a project with the Institute of Medicine (now the National Academy of Medicine) to define operational steps that could be taken by the mental health and substance use fields to implement integrated care. The Institute of Medicine convened a panel that produced a report released in 2006.^11^ This report outlined a series of especially useful practical steps that the field could take to expand integrated care. Much of this report is still current today, especially since integrated care is now beginning to play a key role in the response to the opioid use crisis.^12^

A second major development also occurred in 2006 with the publication of a study by the senior author on premature mortality in persons with mental illness served by public mental health systems.^13^ Based upon data from eight states, the study results showed that these persons died prematurely, on average, 25 years younger than other people in their respective state populations. These findings were shocking to the behavioral health care community, and they served as an important impetus to subsequent legislative and policy developments.

Integrated care was given great boosts by the passage of the Mental Health Parity and Addiction Equity Act of 2008^14^ and the Patient Protection and Affordable Care Act of 2010.^15^ The former provided a legal basis for financial parity between primary care services and mental health and substance use care services. Although major types of insurance were not covered by this Act (i.e., Medicare and Medicaid), most large-scale private insurance plans that offered behavioral health benefits were covered.

The 2010 Affordable Care Act also gave great emphasis to integrated care; it introduced the philosophy of person-centered care—considering the whole person simultaneously and that person’s needs rather than only focusing on a single diagnosis. This new focus also contributed to the subsequent development of self-directed care,^16^ as described below. It also authorized the development of health homes in accountable care organizations through the Medicaid program. These health homes were to offer primary care and behavioral health care simultaneously.^17^

Even though a philosophical basis had been developed and federal financial awards were available for undertaking this work, states, counties, and other providers of behavioral health care services demonstrated relatively little uptake of integrated care. As recollected by the senior author who did considerable work in the field at that time, many behavioral health care providers feared that their care entities would be closed and that they would lose their jobs. Such fears persisted, even in the face of overwhelming evidence that there were far too few behavioral health care providers. At that time, only about one-half of those with behavioral health conditions were being provided any care at all.^18^

Another effect of the 2010 Affordable Care Act was the gradual development of self-directed care through advocacy by the behavioral health consumer community. As a result of this development, behavioral health care providers were encouraged by their clients not only to consider whole-person care but also to deliver it in a manner that promoted joint decision-making between provider and consumer.^16^

The introduction of virtual care allayed some of the fears of behavioral health care service providers that they would lose their jobs or that their organizations would be discontinued. The gradual introduction of virtual care between 2015 and 2020 accelerated the implementation of integrated care. With virtual care through the internet, integrated care could be provided either by a behavioral health provider or a primary care provider, almost anywhere, and without the need to collapse the organizations that each type of provider represented. Telehealth use for behavioral health services has increased 45 times since the start of the COVID-19 pandemic across all care settings and provider types. Less than 1% of all behavioral health visits were delivered via telehealth pre-pandemic. By the second quarter of 2022, 32.8% of behavioral health appointments were conducted through telehealth.^19^ Like the original introduction of virtual care, this step again accelerated the movement toward integrated care.

## Current Developments

The onset of COVID-19 in March 2020 and its subsequent effects have had a major impact on behavioral health care and the evolution of integrated care. In the COVID-19 period, many families were sequestered in their own homes for a considerable period of time. Adults worked from home using the internet, and children attended school from home using the internet. These restrictions had a major impact on the growing prevalence of behavioral health conditions during this period.^20^ At the height of COVID-19, the prevalence of behavioral health conditions in the US population had approximately doubled from its earlier levels of one in four. This meant that almost one in every two adults and children had at least one behavioral health condition at the height of the pandemic.^20^

COVID-19 also had an impact on decreasing the availability of behavioral health care providers. Due to burnout and threats of contracting COVID-19, some behavioral health providers chose to leave the field in this period.^21^ The combination of fewer providers and a prevalence that had almost doubled exacerbated the human resource crisis in behavioral health care, which had been developing gradually for a quarter century.

These same factors also have had the impact of increasing reliance upon primary care physicians for behavioral health care services and encouraging them to collaborate more closely with the behavioral health care community. Although relatively little has been studied about this process of collaboration, these latter developments likely also have increased the implementation of integrated care.

Another important takeaway from the COVID-19 period has been the critical importance of the social determinants of life in generating trauma and subsequent behavioral health conditions. Much of the growth in the prevalence of behavioral health conditions during the COVID-19 period has been due to the trauma experienced by people as a result of these determinants, e.g., being restricted to one’s home for protracted periods of time. Addressing this problem requires the introduction of new interventions to alter these negative social life determinants. These new procedures are called “social care.” A concerted effort now is underway to develop social care and to implement it.^22^ At its most fundamental level, social care can include social support, housing support, and employment support. To be able to add social care to integrated care in an effective manner will require additional training for most behavioral health care providers. Social care is also discussed below under future developments.

## Implications for Behavioral Health

Below, the authors describe several major actions that behavioral health must take to influence the future evolution of integrated care.

### Address Workforce Issues

An urgent need currently exists to address the human resource crisis in behavioral health. This will require not only the identification and training of a larger number of professionals and allied professionals but also efforts to move from a purely clinical approach to one that also includes community-level interventions.^23^

Part of the human resource crisis can be addressed by identifying people currently in the community who have professional and allied professional credentials to work in the behavioral health care field but are not currently doing so. This could include retirees who may only wish to work or volunteer part-time, parents who left the workforce at the time a child was born but now are ready to return to the workforce, as well as others in the community who have an interest in supporting behavioral health care activities, but who do not have any formal training. Most of the work to identify these people will, out of necessity, be done at the community and county levels.

Changes also will be required in the formal training programs for professionals and allied professionals. Not only is there a need to develop the capacity to train a larger number of persons simultaneously, but also the capacity to streamline training where needed, e.g., shortening the length of a psychiatric residency by adding a short period of supervision once the person is in practice, or shortening the required hours of supervision that psychologists, social workers, and mental health counselors are required to fulfill in order to practice.

For allied professionals, the number of peer supporters must be expanded, and other allied professionals who are currently only marginally involved in behavioral health care must be engaged, e.g., physician assistants, community health workers, and nurse assistants. This likely will require the development of gateway programs for these allied professionals to enter the field. Such programs should include a good introduction to the epidemiology of behavioral health care and the behavioral health care field itself, as well as job opportunities currently available locally and more broadly.

Most of the changes in training will necessitate the intervention and oversight of federal agencies, particularly SAMHSA and HRSA. These federal entities will require appropriate congressional legislation to provide the necessary technical staff and funding to undertake essential human resource development activities. Early in 2024, SAMHSA already has hosted a technical expert panel on behavioral health care workforce issues.^24^

### Develop Community Interventions

Community-level interventions will need to be developed to reduce the traumas that lead to behavioral health conditions. These will include efforts to empower communities to develop better functioning structures that provide informal support to people living there. Work of this type already is underway at the Hogg Foundation^25^ in both rural and urban Texas communities. This Hogg Foundation work seeks to empower communities to solve their own problems. The foundation does this by listening to what the community says about its problems, identifying Indigenous leadership in the community, including historically excluded groups, such as particular minorities in the community, and by celebrating community successes when they occur. Beyond broad-based community development, more targeted efforts also will be required to mitigate and remove negative social life determinants which are known to lead to behavioral health conditions. Such work will require skills in community organization and public health, as well as better collaboration with both of these fields by those working in behavioral health care.

### Implement Population Health Management

Even as work is being undertaken to mitigate or remove negative social life determinants, simultaneous efforts must be made to implement population health management strategies. Such strategies can be used to identify community members who are negatively affected by their social determinants of life. This will permit efforts to engage in preventive interventions for people exposed to these determinants. The goal is to intervene early to prevent a subsequent behavioral health crisis from occurring.

The latter effort to engage with persons who are exposed to particular social life determinants is the primary mission of social care. As noted earlier, much more effort must be put into social care. Ideally, this will involve consideration of addressing social care needs for all clients receiving integrated care. Incorporating social care into more traditional integrated care will require new training for many behavioral health care providers. This training will involve the development of skills in working with a consumer who has been exposed to a particular negative social life determinant, addressing the sequelae of that exposure, and actually altering the negative determinant. Many current behavioral health care providers do not have these skills today.

The field of social work is playing a leading role in the development of social care.^22^ This work includes greater operational specification of the particular features of social care and the development of strategies to implement social care in current behavioral health care and health care programs. Such developments can be expected to grow over the next decade.

### Implement the Innovation in Behavioral Health Model

Just very recently, CMS has taken a crucial step to improve the delivery of integrated care while also combining it with social care.^26^ This new program, the Innovation in Behavioral Health (IBH) Model, aims to improve the quality of care and health outcomes for people with moderate to severe behavioral health conditions, including mental health conditions and substance use disorders. The model will support Medicaid and, optionally, Medicare recipients in community-based behavioral health care practices to receive whole-person care in a behavioral health care setting. Behavioral health providers will lead a care management team, coordinating with other providers to best serve beneficiaries. The care management team will address behavioral and physical health issues, as well as health-related social needs, such as housing and food insecurity. The payment model for this new program will follow Medicaid for recipients from that program and value-based purchasing for Medicare beneficiaries, which emphasizes quality over quantity of care.

Here is how CMS envisions that the IBH Model will work: “Community-based behavioral health practices…will be responsible for conducting screenings and assessments of behavioral and physical health, and health-related social needs, offering treatment as appropriate within their scope of practice, providing “closed-loop” referrals to other primary care providers, specialists, and community-based resources, and monitoring ongoing conditions. Since people with moderate to severe behavioral health conditions frequently visit behavioral health settings, this approach uses the behavioral health setting as a point of entry to identify and secure further care and facilitate close collaboration with primary and specialty care providers.”^26^ (2024:1–2).

CMS will release a Notice of Funding Opportunity in the Spring of 2024. Up to eight states and territories will be invited to participate on a voluntary basis. The IBH Model will launch in the Fall of 2024 and run for eight years.

### Improve Reimbursement Rates

An urgent need also exists to continue to improve reimbursement rates for behavioral health care providers delivering integrated care, as well as to expand the range of providers eligible for these enhanced reimbursement rates. In its roadmap for behavioral health integration,^27^ HHS reported that, early in 2024, Medicare had expanded reimbursement for behavioral health care and that CMS had released new guidance for reimbursement for interpersonal consultation. Both of these changes aim to improve integrated behavioral health care.^27^ However, as reported by the National Association of Social Workers, more work remains to expand reimbursement rates for social workers.^28,29^ Within the past year, federal legislation was passed, and CMS implemented expanded coverage for clinical mental health counselors and marriage and family therapists. However, social workers were excluded from this new legislation.^28,29^

### Explore AI

As the field moves further toward universally available integrated care, it will be important to explore the role that artificial intelligence (AI) may be able to play. Recent developments already show that AI can reduce the workload for primary care physicians.^30–32^ Some examples of the use of AI in health care include, using AI algorithms to analyze medical imaging data to quickly determine and accurately diagnose, as well as utilizing Automatic Speech Recognition (ASR) to convert spoken language to written text, streamlining the documentation process. Similar explorations will need to be undertaken for behavioral health care.

## Conclusion

Despite its critical importance for many behavioral health care clients, the path to implementation of integrated care has been quite long and difficult. It also is clear that a number of the impediments to the implementation of integrated care will persist into the future. Thus, the behavioral health care field must take additional steps to assure that integrated care is available to those who require it. The authors encourage the leaders of behavioral health care to undertake a national convening to take stock of developments to date and to outline critical next steps toward better implementation in the future. The authors have tried to outline some of these key future steps here.

As a final note, the authors also would propose that the behavioral health care field consider adopting a new name for integrated care. The integrated care practiced today is far different than that begun almost fifty years ago. The current terminology puts the primary focus upon the providers involved rather than upon the recipient or the care being delivered. Thus, the field might wish to consider terms such as “whole-person care,” “person-centered care,” or something similar to redirect this emphasis.

## References

[1] Osborn DP. The poor physical health of people with mental illness. *The Western Journal of Emergency Medicine*. 2001;175(5):329-332. 10.1136/ewjm.175.5.329. 10.1136/ewjm.175.5.329 PMC1071612 11694483

[2] Auerswald EH. The Gouverneur Health Services Program: An experiment in ecosystemic community health care delivery. *Family Systems Medicine* 1983; 5–24.

[3] Coleman JV, Patrick DL, Baker SM. The mental health of children in an HMO program. *The Journal of Pediatrics* 1977; 150–153. 10.1016/s0022-3476(77)80469-1 874653

[4] McCarrick AK, Manderscheid RW, Bertolucci DE, et al. Chronic medical problems in the chronically mentally ill. *Hospital and Community Psychiatry*. 1986;37(3):289-91. 3957277 10.1176/ps.37.3.289

[5] Regier DA, Burke JD, Manderscheid RW, et al. The chronically mentally ill in primary care. *Psychological Medicine.* 1985;15(2):265-273. 10.1017/S003329170002354. 4023131 10.1017/s0033291700023540

[6] Manderscheid, R.W. Formulation of mental health policy in the United States, with comparative case studies of South Africa and Thailand. In E.S. Sorel, (Ed.) *21*^*st*^* Century Global Mental Health* Burlington, MA: Jones and Barlett Learning; 2006. pp. 351-364

[7] Satcher D. *Mental Health: A Report of the Surgeon General*. Rockville MD: U.S. Department of Health and Human Services, 1999.

[8] Office of the Surgeon General. *Report of a Surgeon General's Working Meeting on The Integration of Mental Health Services and Primary Health Care*. Rockville, MD: Office of the Surgeon General 2001. https://www.ncbi.nlm.nih.gov/books/NBK44335/. Accessed 18 June 2024. 20669517

[9] Roby DH, Jones EE. Limits on same-day billing in Medicaid hinders integration of behavioral health into the medical home model. *Psychological Services.* 2016;13(1):110-119. 10.1037/ser0000044. 26845494 10.1037/ser0000044

[10] The Kennedy Forum. *Mental Health Advocacy*. (2024, February 29). https://www.thekennedyforum.org/mental-health-advocacy/. Accessed 18 June 2024.

[11] Committee on Crossing the Quality Chasm, Institute of Medicine. *Adaptation to Mental Health and Addictive Disorders. Improving the Quality of Health Care for Mental and Substance-Use Conditions: Quality Chasm Series*. Washington, DC: National Academies Press; 2006. 10.17226/11470. 20669433

[12] Alcohol, Tobacco, and Other Drugs Section. *Integrated Behavioral Health Spotlight: Utilizing IBH to increase access to opioid use disorder care.”* Webinar presented by Drs. Blake Fagan and Zach White, American Public Health Association. (2024, June 4).

[13] Colton, C.W., & Manderscheid, R.W. Congruencies in increased mortality rates, years of potential life lost, and causes of death among public mental health clients in eight states. *Preventing Chronic Disease*, 2006: 3(2), A42. Accessed July 16^th^, 2021, from http://www.cdc.gov/pcd/issues/2006/apr/05_0180.html. PMC1563985 16539783

[14] United States Congress. (2008). *Mental Health Parity and Addiction Equity Act,* Public Law NO. (2008):110–343, 122 Stat. 3765.

[15] United States Congress*. Patient Protection and Affordable Care Act*, Public Law 148, U.S. Statutes at Large 124 (2010):*119–1024*. https://www.govinfo.gov/app/details/STATUTE-124/STATUTE-124-Pg119/summary. Accessed 18 June 2024.

[16] Human Services Research Institute. *Demonstration and Evaluation of Self-Direction in Mental Health*. No date. https://www.hsri.org/project/demonstration-and-evaluation-of-self-direction-in-mental-health. Accessed 18 June 2024.

[17] Centers for Medicare & Medicaid Services. *Health Homes Overview.* No date. https://www.medicaid.gov/medicaid/long-term-services-supports/health-homes/index.html. Accessed 18 June 2024.

[18] Wang PS, Lane M, Olfson M, et al. Twelve-month use of mental health services in the United States: Results from the National Comorbidity Survey Replication. *Archives of General Psychiatry*. 2005;62(6):629-640. 10.1001/archpsyc.62.6.629. 15939840 10.1001/archpsyc.62.6.629

[19] Coe E, Enomoto K, Lewis R. *Telehealth: A Post-COVID-19 Reality?* McKinsey & Company. Published May 29, 2020. Accessed May 31, 2024. https://www.mckinsey.com/industries/healthcare/our-insights/telehealth-a-post-covid-19-reality.

[20] Centers for Disease Control. *Household Pulse Survey.* 2021.https://www.cdc.gov/nchs/covid19/health-care-access-and-mental-health.html.

[21] Lluch C, Galiana L, Doménech P, Sansó N. The impact of the COVID-19 pandemic on burnout, compassion fatigue, and compassion satisfaction in healthcare personnel: A systematic review of the literature published during the first year of the pandemic. *Healthcare*. 2022; 10(2):364. 10.3390/healthcare10020364. 35206978 10.3390/healthcare10020364 PMC8872521

[22] Arndt-Couch, RMA. *Institute on Social Practice Integration Research and Education (INSPIRE): A Workforce Development Solution to Close the Health Gap.* Los Angeles. University of Southern California Libraries. 2023. https://digitallibrary.usc.edu/asset-management/2A3BF1MSKPU7K?WS=SearchResults. Accessed 18 June 2024.

[23] Manderscheid R, & Ward A. Stepping into the future of mental health. *Journal of Orthopsychiatry*. In press. 10.1037/ort0000743 38661652

[24] Manderscheid R. *SAMHSA Takes Action to Address Our Workforce Crisis.* https://www.hmpgloballearningnetwork.com/site/bhe/perspectives/samhsa-takes-action-address-our-workforce-crisis. Accessed 18 June 2024. Published January 24, 2024.

[25] Hogg Foundation, *Transforming Mental Health Through Community*. No date. https://hogg.utexas.edu/. Accessed 18 June 2024.

[26] Centers for Medicare & Medicaid Services. *Innovation in Behavioral Health (IBH) Model.* 2024. https://www.cms.gov/priorities/innovation/innovation-models/innovation-behavioral-health-ibh-model. Accessed 18 June 2024.

[27] United States Department of Health and Human Services. *HHS Roadmap for Behavioral Health Integration Fact Sheet: Accomplishments*. Published January 18, 2024. Accessed June 5, 2024. Available from: https://www.hhs.gov/about/news/2024/01/18/hhs-roadmap-for-behavioral-health-integration-fact-sheet-accomplishments.html.

[28] National Association of Social Workers. *Reimbursement*. No date. (https://www.socialworkers.org/Practice/Clinical-Social-Work/Reimbursement. Accessed 18 June 2024.

[29] National Association of Social Workers. *NASW leads sign-on letter of 64 diverse national organizations in support of the Improving Access to Mental Health Act* (S. 838/H.R. 1638). November 2, 2022. https://www.socialworkers.org/Advocacy/Policy-Issues/Medicare-Reimbursement#:~:text=Further%20The%20Improving%20Access%20to,schedule%2C%20thereby%20mitigating%20reimbursement%20inequity. Accessed 18 June 2024.

[30] Los Angeles Pacific University. *Revolutionizing Healthcare: How is AI being Used in the Healthcare Industry?* Published December 21, 2023. Accessed May 31, 2024. https://www.lapu.edu/ai-health-care-industry/.

[31] Shamszare H, Choudhury A. Clinicians' perceptions of artificial intelligence: Focus on workload, risk, trust, clinical decision making, and clinical integration. *Healthcare.* 2023; 11(16):2308. 10.3390/healthcare11162308. 37628506 10.3390/healthcare11162308 PMC10454426

[32] Spivey A. *Artificial Intelligence in Health Care: Promise and Pitfalls.* Duke University School of Medicine. Published November 13, 2023. Accessed May 31, 2024. https://medschool.duke.edu/news/artificial-intelligence-health-care-promise-and-pitfalls.

